# A part per trillion isotope ratio analysis of ^90^Sr/^88^Sr using energy-filtered thermal ionization mass spectrometry

**DOI:** 10.1038/s41598-022-05048-7

**Published:** 2022-01-21

**Authors:** Shigeyuki Wakaki, Jo Aoki, Ryoya Shimode, Katsuhiko Suzuki, Takashi Miyazaki, Jenny Roberts, Hauke Vollstaedt, Satoshi Sasaki, Yoshitaka Takagai

**Affiliations:** 1 Kochi Institute for Core Sample Research, Japan Agency for Marine-Earth Science and Technology (JAMSTEC), 200 Monobe Otsu, Nankoku, Kochi 783-8502 Japan; 2 Faculty of Symbiotic Systems Science, Cluster of Science and Technology, Fukushima University, 1 Kanayagawa, Fukushima, 960-1296 Japan; 3 Submarine Resources Research Center, JAMSTEC, 2-15 Natushima, Yokosuka, Kanagawa 237-0061 Japan; 4 Volcanoes and Earth’s Interior Research Center, JAMSTEC, 2-15 Natushima, Yokosuka, Kanagawa 237-0061 Japan; 5 Thermo Fisher Scientific Bremen GmbH, Hanna-Kunath-Str. 11, 28199 Bremen, Germany; 6 Thermo Fisher Scientific K.K, 4-2-8 Shibaura, Minato-ku, Tokyo, 208-0023 Japan; 7 Institute of Environmental Radioactivity, Fukushima University, 1 Kanayagawa, Fukushima, 960-1296 Japan

**Keywords:** Mass spectrometry, Environmental monitoring, Nuclear chemistry

## Abstract

Strontium-90 is a major radioactive nuclide released by nuclear accidents and discharge waste. Input of such radioactive nuclide into earth surface environment causes potential threat of long-term internal exposure when taken up by organism. Rapid and precise measurement of ^90^Sr in variety of environmental sample is important to understand the distribution and dynamics of ^90^Sr in the local environment after the accident and to assess the effect of radioactive nuclide inputs on bodies. However, previous ^90^Sr measurement techniques have drawbacks such as long measurement times for radiometry and high detection limits for mass spectrometry. Here we present a technique to accurately measure a significantly small amount of ^90^Sr in natural environmental samples using an energy-filtered thermal ionization mass spectrometry. Our technique achieved a ^90^Sr detection limit of 0.23 ag, which corresponds to a ^90^Sr activity of 1.2 µBq. The detection limit was lowered by two orders of magnitude compared with the previous mass spectrometric ^90^Sr analyses. The ability of our technique will expand the applicability of mass spectrometric ^90^Sr survey not only to the rapid ^90^Sr survey upon nuclear accidents but also to study a long-term environmental diffusion of radioactive materials using size-limited environmental and biological samples.

## Introduction

The moment of history or dynamics of human and environment are precisely memorized as isotope ratios in variety of materials. Understanding the isotope ratio stem from radioactive isotope can realize an aspect of past condition and transition. Among the isotope ratio, that of strontium (Sr) reflects past aspect and/or a fact basing on radioactive decay or mass discrimination effect, and it widely serves indictors as age determination in geological sciences^1^, environmental dynamics^2,3^, forensic sciences^4^, authenticity judgment^5^, and determination of geographic origin^6^. In particular, the artificial nuclide, radioactive strontium (^90^Sr) records as the memory of nuclear disaster in materials such as Chernobyl or Fukushima Nuclear Power Plant Accidents^7^.

^90^Sr is a β-decay only radionuclide (0.55 MeV, 100% yield) and a typical fission product of ^235^U (5.8% accumulated fission yield) and ^239^Pu (2.1% accumulated fission yield). Anthropogenic ^90^Sr is widely spread over earth surface environments with low background radioactivities because of the use and testing of nuclear weapons^8^. Nuclear accidents are another source of anthropogenic ^90^Sr emissions into the environment, resulting in local fallout with high activity concentrations with 1.4–80.8 Bq/kg^9^. ^90^Sr was one of the major radioactive nuclides released in the Chernobyl^10–12^ and Fukushima^9,13–15^ nuclear reactor accidents. When taken up by animals, including humans, ^90^Sr has the potential to cause long-term internal exposure^16,17^. The slight trace of ^90^Sr is precisely memorized as an isotope ratio between stable isotope of Sr in variety of samples^18^. Therefore, a precise measurement the isotope ratio of ^90^Sr and stable Sr is important to determine the understanding of right dynamics of ^90^Sr in the local environment surrounding the accident site^19^.

Conventionally, radiometric methods using solid/liquid scintillators or gas ionization detectors were used to detect ^90^Sr in various environmental samples^5,12–15^. The radiometric method is fundamentally a counting experiment of the decaying atoms. Because ^90^Sr has a half-life of 28.79 years^24^, the fraction of the decaying atom is significantly small: approximately 1.3 × 10^9^ atoms of ^90^Sr corresponds to 1 Bq (i.e. decay per second) of activity. Therefore, for precise radiometric ^90^Sr determination, a relatively large sample size or long analysis time is required^12,17^. For example, 100 g of soil samples are measured by nitrate precipitation-low background gas-flow counting method (nitrate precipitation-LBC) with 2 weeks^9^, and 1 L of seawaters are measured by gross beta radiometric counting with iron-barium co-precipitation method^14^. Because ^90^Sr is a β-particle emitter, radiometric determination of ^90^Sr includes complex radiochemical procedures and typically requires 2 weeks or more measurement time^17,18^. This slow sample processing speed is insufficient for an urgent environmental survey in response to a nuclear emergency^27^.

An alternative and less time-consuming method to analyze ^90^Sr is mass spectrometry. Inductively coupled plasma mass spectrometry (ICP-MS) is a new mass spectrometric technique for ^90^Sr detection^27–31^. Unlike radiometry, the target of mass spectrometric measurement is all the existing ^90^Sr atoms in the sample. The large difference in target numbers suggests that mass spectrometric methods are more sensitive for detecting ^90^Sr. However, measuring a minor isotope is a challenging task in mass spectrometry. Mass spectrometry’s sensitivity to detect trace ^90^Sr is hindered by high noise signals due to isobaric interferences of ^90^Zr and molecular ions, as well as peak tailing of the highly abundant ^88^Sr ions. ICP-MS’s major source of noise signals is ^90^Zr^2,24,25^. In environmental samples, the ratio between minor ^90^Sr and more abundant stable isotopes of Sr or isobaric isotope ^90^Zr is extremely high (i.e., at least nine orders of magnitude^15^). ICP ion source efficiently ionizes ^90^Zr, which remains in the sample solution in trace amounts even after Sr extraction chemistry. The reaction of the ions with O_2_ gas is used in a dynamic reaction cell (DRC) technique to reduce the ^90^Zr ion transmission^2,24,25^. Typically, this technique achieves abundance sensitivities, defined as the intensity ratio between ^88^Sr peak tail on m/z = 89.908 and ^88^Sr, on the order of 10^−9 ^^2,19^. Furthermore, the introduction of a new technique, triple quadrupole ICP-MS (ICP-MS/MS), combined with the O_2_ gas reaction, effectively reduced the ^90^Zr ion transmission in the mass spectrometer and lowered the noise signal to 0.1 cps, which correspond to a ^90^Sr detection limit of 0.11 fg (0.6 mBq)^28^. With a sample size of 4 µg of Sr, the abundance sensitivity for the ^90^Sr/^88^Sr ratio achieved by this technique was 5 × 10^−12 ^^28^. However, reducing the ^90^Sr detection limit with the ICP ion source is difficult. The highly efficient ICP ion source mainly produces abundant polyatomic or polyvalent ions from the sample solution’s solvents, Ar gas, and trace impurities in the sample solution, and such ions exist across the entire m/z range. It also emits Ar gas-related ions such as Ar^+^ and ArO^+^ at significantly high intensities, which may cause non-spectrum interference of the peak tails. All such interferences are increasing the noise level and limiting the sensitivity of ICP-MS analysis. Even with the ICP-MS/MS technique, the background signal was reported as 0.1–0.2 cps when aspirating a blank solution^28^, showing that noise signals of sub-cps level are inevitable while using the ICP ion source.

Thermal ionization mass spectrometry (TIMS) is a standard technique for measuring isotope ratios of Sr, such as ^87^Sr/^86^Sr and ^88^Sr/^86^Sr^34^. Compared with the ICP ion source, the thermal ionization ion source is energy-limited, and thus ionization is mostly limited to target elements with very few polyatomic ions. In TIMS, Ar-gas-related species, solvent-related species, and polyvalent ions are mostly absent. With less spectrum and non-spectrum interferences, TIMS can reduce noise signal levels on ^90^Sr and thus increase sensitivity upon ^90^Sr detection. Recently, several attempts have been made to detect ^90^Sr using the TIMS technique. Previous studies have failed to detect traces of ^90^Sr in environmental samples^27,28^. Kavasi et al*.* measured ^90^Sr/^88^Sr ratios of ^90^Sr-containing reference materials of wild berry and lake sediment using a sample size of 1000 ng of Sr and reports an abundance sensitivity for the ^90^Sr/^88^Sr ratio as 2.1 × 10^−10^, corresponding to a ^90^Sr noise signal of 0.77 cps^37^. However, when the ^90^Sr/^88^Sr ratio is lower, their measured ^90^Sr/^88^Sr ratio shows a systematic bias toward higher values. To account for such a significant bias, additional empirical “relative bias” errors must be introduced into the analytical uncertainty. This inaccuracy is obvious when the ^90^Sr/^88^Sr ratio is lower than 1.2 × 10^−9^ and is likely to be caused by an inaccurate noise correction scheme for the ^90^Sr signal. Ito et al*.* focused on analyzing small-sized samples and constructed an isotope-dilution total-evaporation (ID-TE-) TIMS technique to analyze ^90^Sr with a sample size of 5–20 ng of Sr^38^. Their study did not report abundance sensitivity, but it can be estimated as 2 × 10^−8^ based on the reported analytical conditions of ^90^Sr noise level of approximately 5 cps and ^88^Sr target intensity of 4 V^38^. Among the mass spectrometric ^90^Sr analysis methods, TIMS is the only method that has been confirmed by an independent IAEA proficiency test^39^. During the TIMS analysis, the ionization of ^90^Zr^+^ is suppressed, whereas Sr is still present and ionizing from the filament^38^ because of the difference in the evaporation and ionization potentials between the two elements. Therefore, the peak tailing of the highly abundant ^88^Sr ions is the main source of background signal for ^90^Sr in TIMS measurements. Peak tail ions can be efficiently reduced using an energy filtering device placed immediately before the detector. However, none of the previous ^90^Sr analyses used an effective energy filtering device to account for the ^88^Sr tailing^35–38^. Kavasi et al*.* used a WARP energy filter. However, they did note that the WARP energy filter eliminates low energy ions but has no effect on high-mass side peak tailing, implying that the WARP energy filter does not work for ^88^Sr peak tail on ^90^Sr. The ability of TIMS to detect a significantly small amount of ^90^Sr remains unknown.

In this study, we focused on bringing out the performance of modern TIMS instruments to perform an accurate ^90^Sr/^88^Sr measurement of environmental and biological samples with low ^90^Sr activity using an effective energy filtering technique for ^90^Sr detection. The Retarding Potential quadrupole (RPQ) lens act as high selectivity filter for ions with disturbed energy or angle^40^. The use of RPQ lenses for energy filtering coupled with reduction of the multiple noise signal sources resulted in a significantly low and highly stable noise signal for ^90^Sr compared with the previous studies. With significantly low and stable noise signals, an appropriate noise correction scheme was used in this study to allow accurate measurement of ^90^Sr/^88^Sr ratio down to 10^−11^ level. The measurement of ^90^Sr-containing reference materials (plants), as well as environmental and biological samples, demonstrates the ability of our technique.

## Results and discussion

### Noise reduction schemes upon ^90^Sr detection by energy-filtered TIMS

**Figure 1:**
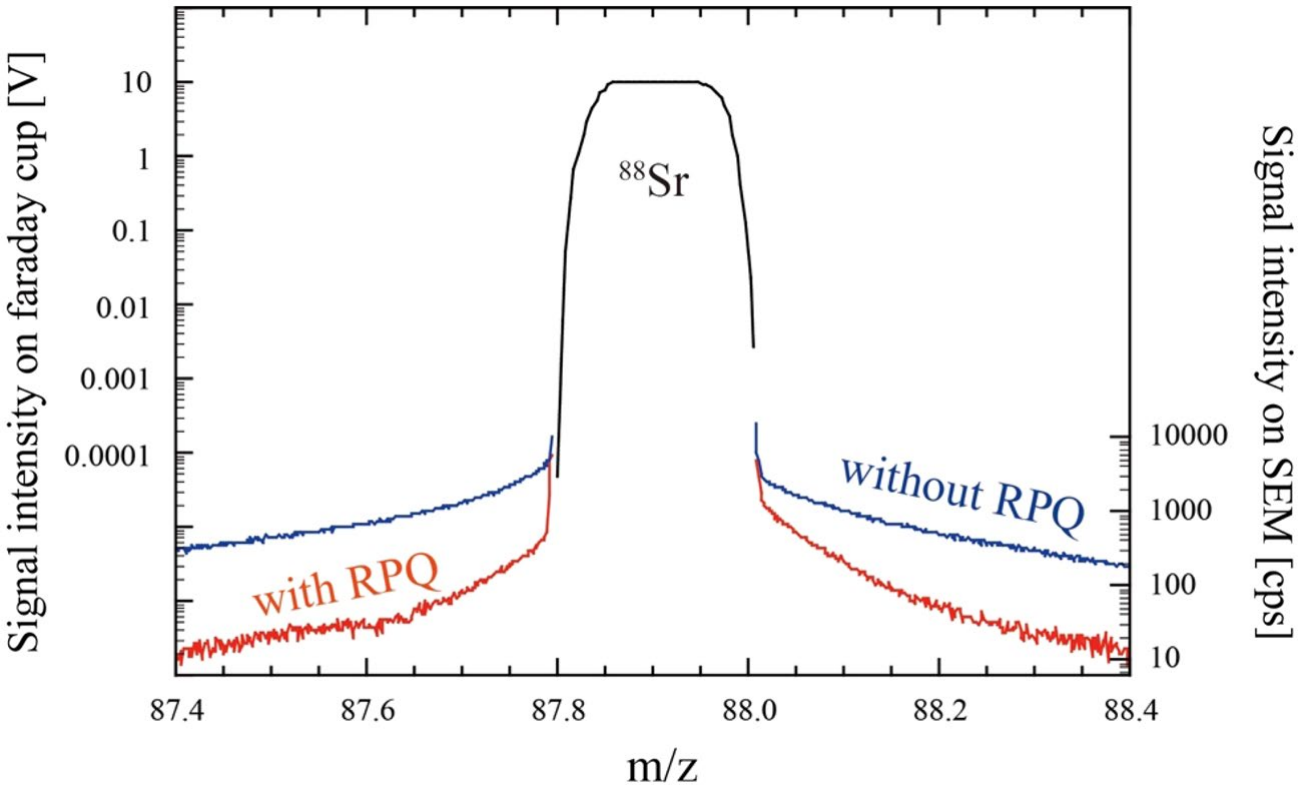
Mass spectrum in the vicinity of m/z = 88 showing the flat-top peak of ^88^Sr measured by the center Faraday cup (black lines). The ^88^Sr peak tails on the low- and high-mass sides is also shown. Peak tails measured by SEM without the RPQ are shown in blue lines and peak tails measured by SEM with the RPQ are shown in red lines.

Peak tailing of ^88^Sr, which has the largest abundance among Sr isotopes, is the main limiting factor in TIMS detection of ^90^Sr. The RPQ lenses used in this study eliminate peak tailing from the high-mass and low-mass sides of the ^88^Sr peak (Fig. 1). The RPQ lens parameters must be fine-tuned to effectively eliminate the ^88^Sr peak tailing on ^90^Sr while sacrificing only a small amount of ion transmission rate and peak shape (Figure S1 in supporting information). Finally, after the introduction and fine-tuning of the RPQ lenses, a 1000-fold reduction of the ^88^Sr peak tail signal was achieved (Figure S2 in supporting information).

Because the ^88^Sr peak tail signal has been reduced to approximately 0.01 cps on ^90^Sr, signals from other noise sources must also be controlled at this noise level. The filament material, rather than the sample-derived Zr, is the major source of the isobaric ^90^Zr signal in TIMS measurement. Typically, filaments were baked out at 4500 mA (approximately 2000 °C) before the use. With this level of bake out, about 1 cps and about 0.1 cps of the ^90^Zr^+^ signals were observed from Re and Re-ZR filaments, respectively, during Sr isotope ratio measurement even with the presence of Sr sample on the filament. These were not acceptable levels of noise in this study. Therefore, filaments were baked at higher temperatures at a 5.5 A filament current (approximately 2200 °C) to reduce the filament-derived Zr^+^ signal. After this high temperature bakeout, the Re and the Re-ZR filaments tend to show ^90^Zr^+^ signals on the 0.01 cps order when heated up to 1550 °C without the sample material. Even after this high temperature bakeout, a few Re and Re-ZR filaments still demonstrate higher ^90^Zr^+^ emission. Therefore, after the bakeout, all the filaments were inspected for ^90^Zr intensity to ensure that filaments with higher ^90^Zr^+^ emission were not used. The ^90^Zr intensity threshold at 1550 °C was empirically set to 0.04 cps. Note that the actual ^90^Zr intensity during Sr isotope measurement is negligible (far lower than 0.04 cps) because Zr^+^ emission is suppressed during the presence of Sr on the filament.

Ito et al. observed that organic material with m/z 90.0 is a significant isobaric interfering molecule on ^90^Sr, and this could be perfectly mass separated by slightly shifting the axial m/z from 89.908 to 89.777^38^. We detected another isobaric interference molecule spectrum at m/z 89.908 with intensities on the order of 0.1 cps or less (Figure S3 in supporting information). The intensity of this noise signal is relatively high at the beginning of the measurement and decreases with time (Figure S4 in supporting information), indicating that this noise signal is related to organics or volatiles. The most plausible molecule for this mass spectrum is ^88^SrH_2_^+^, with hydrogen derived from organics or residual H_2_O. This noise signal was eliminated by preheating of the sample filament under vacuum before the measurement and by avoiding the use of parafilm/catheter during sample loading.

### Abundance sensitivity and detection limit of the ^90^Sr/^88^Sr measurement

**Figure 2:**
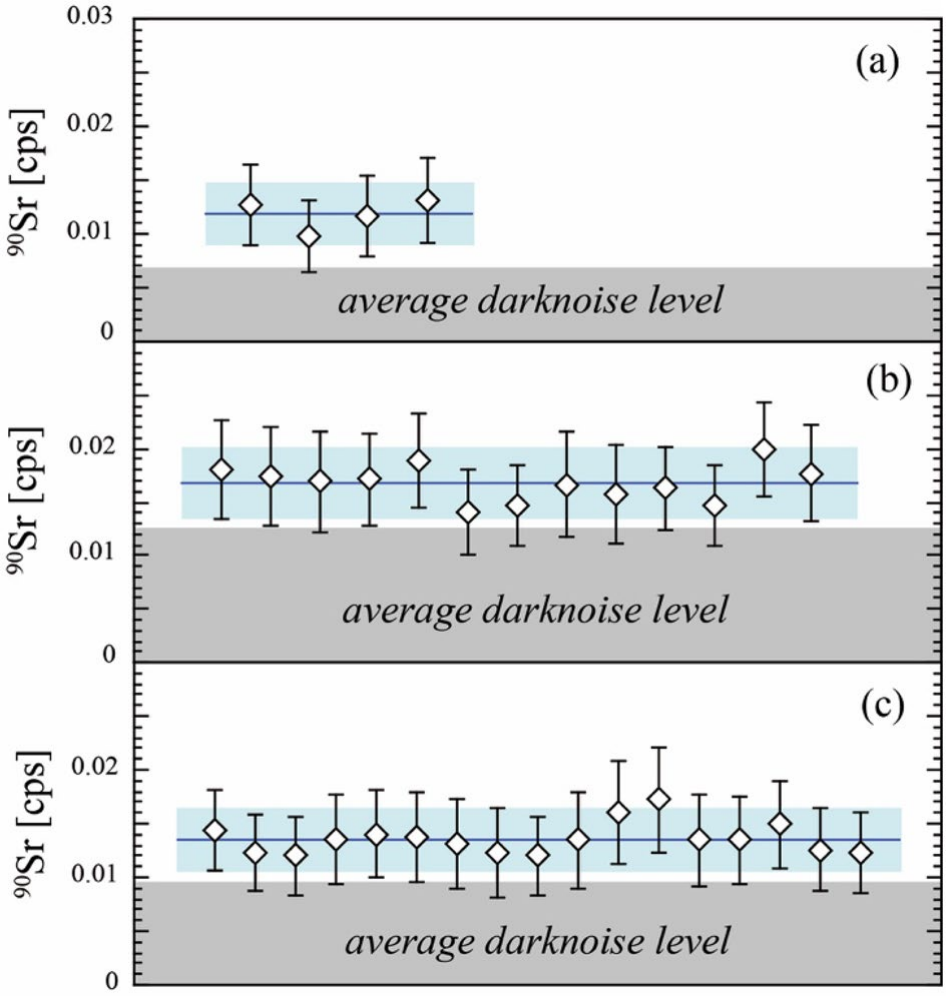
Results of repeated ^90^Sr measurements of ^90^Sr-free NIST SRM987 in (**a**) Bremen, (**b**) Fukushima-1, and (**c**) Fukushima-2 sessions. White diamonds represent noise uncorrected data of a single measurement. Blue line and blue bands represent the average and 2 SD range of the multiple measurements. The grey area represents the average intensity of dark noise observed during each measurement session.

The ^90^Sr-free NIST SRM-987 was measured using all these noise reduction schemes to validate the noise level and noise stability on ^90^Sr. The results of the three analytical sessions with slightly different analytical conditions are summarized in Table 1, Fig. 2, and Figure S5 in supporting information.

**Table 1:** Results of replicate measurements of NIST SRM 987 in three different mass spectrometric sessions.

Session	Reference date	^88^Sr (V)	^90^Sr^1^^,2^ (cps)	Average darknoise (cps)	^90^Sr/^88^Sr^1,2^ Abundance sensitivity	3SD detection limit	Number of analysis
Bremen	January 22nd, 2020	20.3	0.0118 (29)	0.0070	9.3 (3.0) × 10^−12^	3.7 × 10^−12^	4
Fukushima-1	April 3rd, 2021	27.2	0.0168 (34)	0.0125	1.0 (2.2) × 10^−11^	3.1 × 10^−12^	13
Fukushima-2	June 5th, 2021	26.3	0.0136 (29)	0.0096	8.3 (1.8) × 10^−12^	2.7 × 10^−12^	17

^1^Errors in the parenthesis are 2SD.

^2^These data are not corrected for SEM dark noise.

The average intensity of the ^90^Sr noise signal of the three analytical sessions were 0.0118 ± 0.0029 (2SD, n = 4), 0.0168 ± 0.0034 (2SD, n = 13) and 0.0136 ± 0.0029 (2SD, n = 17) for *Bremen*, *Fukushima-1st* and *Fukushima-2nd* sessions, respectively. Note that these noise signals include dark noise counts because they were not corrected. Slightly higher noise intensity of *Fukushima-1st* compared with the *Bremen* and *Fukushima-2nd* reflects the fact that higher SEM operating voltage resulted in higher dark noise level in *Fukushima-1st* session (Table 1). The average dark noise count during the *Fukushima-2nd* session was 0.0096 cps, accounting for 70% of the total noise signal. This indicates that our noise reduction scheme has successfully reduced noise signals other than the detector’s intrinsic dark noise to the level of 0.004 cps (Fig. 2). The small variability of the noise signal indicates that the noise signals are well controlled at this level. In this study, the level and stability of the noise signal achieved are superior compared with the noise signals reported by non-energy filtered TIMS technique^37^ (0.77 ± 0.82 cps, 2SD) and by ICP-MS/MS technique^28^ (0.1 cps). Noted that measurement with such a low signal requires a long measurement time (1 h in this study) to count the substantial number of ions. The stable nature of the TIMS ion source allows long measurement with stable analytical conditions.

The abundance sensitivity and detection limit of the ^90^Sr/^88^Sr ratio can be determined from these data. Finally, the abundance sensitivity (^90^Sr/^88^Sr ratio) achieved in the *Fukushima-2nd* session was 8.3 ± 1.8 × 10^−12^ (2SD, n = 17; Table 1). A detection limit of a signal is defined by 3σ of the variability of the zero-point (or blank) analysis. Therefore, the detection limit of ^90^Sr/^88^Sr ratio finally achieved in the *Fukushima-2nd* session was estimated to be 2.7 × 10^−12^ (Table 1 and Figure S5c in supporting information). The fact that the detection limit is lower than the abundance sensitivity demonstrates TIMS’s excellent noise stability and its good control on ^90^Sr.

### ^90^Sr/^88^Sr measurement of the reference materials and natural samples

Table 2 summarizes the details of the analyzed environmental and biological samples, as well as the ^90^Sr activity parameters found in the literature (more details are shown in Table S1 in supporting information). As a ^90^Sr-free sample, a seawater reference material NASS-6 (Atlantic surface water) issued by the National Research Council Canada and a geochemical reference material JCp-1 (modern coral) issued by the Geological Survey of Japan were analyzed. The ^90^Sr-containing samples analyzed in this study include the International Atomic Energy Agency’s certified reference materials IAEA 156 (clover) and IAEA 330 (spinach). We also analyzed the ash from crayfish and smallmouth bass samples collected in Fukushima prefecture, Japan, after the Fukushima Daiichi Nuclear Power Plant accident. The ^90^Sr activity of these samples was analyzed using a radiometric method and published elsewhere^20^.

**Table 2:** ^90^Sr activity of the environmental and biological samples and results of Sr abundance measurement.

Sample	Sample type	^90^Sr (Bq/kg)	Sr (mg/kg)
NASS-6	Seawater	–	7.48^34^
JCp-1	Coral	–	7271 (218)
IAEA 156	Clover	14.8 (3.0)	33.5 (2.0)
IAEA 330	Spinach	20.1 (4.2)	44.0 (2.6)
Ash 1	Crayfish	110 (8)	1344 (66)
Ash 2	Smallmouth bass	25 (2)	414 (28)

All the errors in the parenthesis are 2 SD.

Table 2 also summarized the measured Sr abundance of the ^90^Sr containing samples. The decay corrected ^90^Sr/^88^Sr reference value of these samples was calculated for each analytical session using this measured Sr abundances together with either the certified ^90^Sr activity, for IAEA 156 and IAEA 330, or the radiometrically measured ^90^Sr activity, for Fukushima samples, respectively.

**Figure 3:**
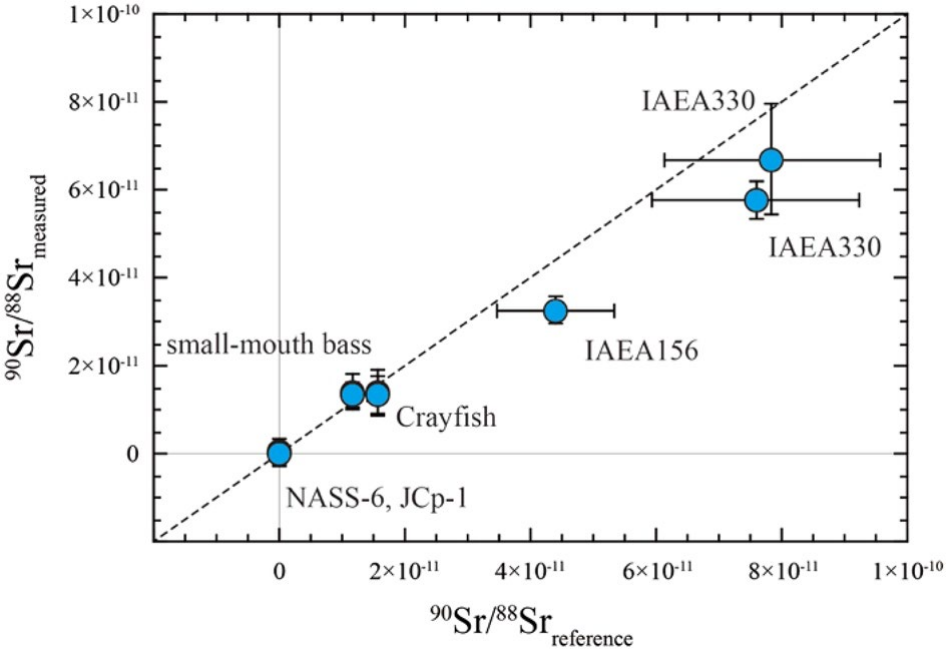
The measured ^90^Sr/^88^Sr ratio of the environmental and biological samples was compared with the decay corrected reference ^90^Sr/^88^Sr ratios. The blue circle represents an average of multiple measurements of the sample. Error bars correspond to 2 SD.

The results of ^90^Sr/^88^Sr ratio measurements of the ^90^Sr containing samples are summarized in Table 3 (more details are shown in Tables S2 and S3 in supporting information), Fig. 3, and Figure S6 in supporting information. The average ^90^Sr/^88^Sr ratios of the ^90^Sr-free samples were − 0.1 ± 2.5 × 10^−12^ (2SD, n = 5), 0.3 ± 2.9 × 10^−12^ (2SD, n = 8) and 0.5 ± 3.4 × 10^−12^ (2SD, n = 5) for NASS-6, JCp-1 (*Fukushima-1*) and JCp-1 (*Fukushima-2*), respectively. These results show that zero values of the ^90^Sr/^88^Sr ratios were measured without any systematic bias. No effect of sample-derived ^90^Zr was observed down to 0.004 cps level. These results demonstrate the excellent stability of the noise signal, as well as the robustness of our noise correction scheme.

**Table 3:** Results of ^90^Sr/^88^Sr measurements of the environmental and biological samples.

Sample	Reference ^90^Sr/^88^Sr^1^	^90^Sr/^88^Sr^2^
NASS-6	–	− 0.1 (2.5) × 10^−12^
JCp-1	–	0.3 (2.9) × 10^−12^
	–	0.5 (3.4) × 10^−12^
IAEA 156	4.4 (0.9) × 10^−11^	3.26 (0.30) × 10^−11^
IAEA 330	7.9 (1.7) × 10^−11^	6.7 (1.3) × 10^−11^
	7.6 (1.7) × 10^−11^	5.76 (0.43) × 10^−11^
Ash 1	1.57 (0.15) × 10^−11^	1.38 (0.53) × 10^−11^
	1.56 (0.15) × 10^−11^	1.32 (0.42) × 10^–11^
Ash 2	1.16 (0.11) × 10^−11^	1.40 (0.39) × 10^–11^
	1.15 (0.11) × 10^−11^	1.33 (0.27) × 10^–11^

^1^Decay corrected to the reference date (see Table S2 in supporting information).

^2^Noise corrected values using the average ^90^Sr count rate of NIST SRM 987 measurements (see text for details). All the errors in the parenthesis are 2 SD.

**Figure 4:**
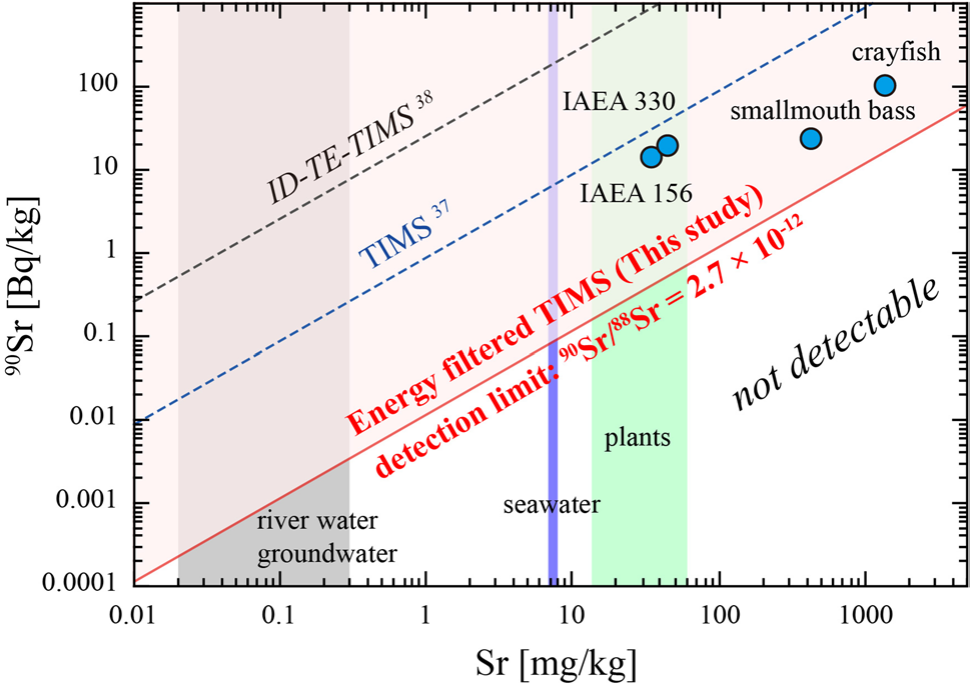
Stable Sr and ^90^Sr abundances of the analyzed samples (blue circles) were compared with the ^90^Sr/^88^Sr detection limit (red line) of this study. The ^90^Sr/^88^Sr detection limit of the previous TIMS studies was plotted for comparison. Stable Sr abundance range of selected environmental samples is also plotted: plants, seawater, and river water/groundwater.

The measured ^90^Sr/^88^Sr ratios of the IAEA 330 were 6.7 ± 1.3 × 10^−11^ (2SD, n = 4) and 5.76 ± 0.43 × 10^−11^ (2SD, n = 5) for temporally separated *Bremen* and *Fukushima-2nd* sessions. Both values agree well with the decay corrected reference ^90^Sr/^88^Sr ratios within analytical errors. The measured ^90^Sr/^88^Sr ratios of the IAEA 156 were 3.26 ± 0.30 × 10^−11^ (2SD, n = 6) and it agrees well with the decay corrected reference value within analytical errors. The measured ^90^Sr/^88^Sr ratios of the Ash1 (crayfish sample) was 1.38 ± 0.53 × 10^−11^ (2SD, n = 6) and 1.32 ± 0.42 × 10^−11^ (2SD, n = 6) for *Fukushima-1st* and *Fukushima-2nd* sessions, respectively. The measured ^90^Sr/^88^Sr ratios of the Ash2 (smallmouth bass sample) were 1.40 ± 0.39 × 10^−11^ (2SD, n = 6) and 1.33 ± 0.27 × 10^−11^ (2SD, n = 6) for *Fukushima-1st* and *Fukushima-2nd* sessions, respectively. These measured values agree well with the decay corrected reference ^90^Sr/^88^Sr ratios within analytical errors (Table 3). No systematic bias exceeding the analytical uncertainties is observed for these results. All these measurements demonstrate the analytical capability of the energy-filtered TIMS technique, as well as our Sr separation chemistry to measure ^90^Sr/^88^Sr ratios of 10^−11^ range (and possibly in the 10^−12^ range) in natural environmental and biological samples (Fig. 4).

### Performance and applicability of ^90^Sr analysis by energy filtered TIMS

Among the environmental and biological samples analyzed, Sr ion beams run short before 220 cycles in some of the measurements with a sample amount of 100 ng of Sr. Nevertheless, 100 ng measurements mostly show precision indistinguishable with the measurement of larger sample sizes. From these observations, we defined that the minimum amount of sample required for our ^90^Sr/^88^Sr measurement as 100 ng of Sr. This is one of the essential parameters to evaluate the performance of TIMS measurement. Given the minimum sample size of 100 ng of Sr, and the ^90^Sr/^88^Sr ratio detection limit of 2.7 × 10^–12^, the detection limit of the absolute ^90^Sr amount can be estimated as 0.23 ag or 0.0012 mBq. The previous TIMS ^90^Sr measurements have reported a ^90^Sr detection limit of 0.17 fg (or 0.88 mBq)^37^ and 0.029 fg (or 0.15 mBq)^38^. Our technique has succeeded to lower the ^90^Sr detection limit of TIMS by three orders of magnitude.

Based on the applicability of this technique to environmental and biological sample measurements, detectable ^90^Sr activity concentrations in this study are different among samples with different stable Sr abundances (Fig. 4). This is because the TIMS technique measures isotope ratios and thus the detection limit of the ^90^Sr analysis is primarily determined as ^90^Sr/^88^Sr ratio. For example, seawater is one of the typical environmental samples and has a stable Sr abundance of approximately 8 mg/kg. With this energy-filtered TIMS technique, the detection limit of seawater ^90^Sr activity concentration is estimated as 0.09 Bq/kg. The minimum quantity of the seawater sample required for the measurement is as small as 12.3 µL. River water and groundwater have stable Sr abundances range from several tens to several hundreds of ppb. In this case, the detection limit of ^90^Sr activity concentration will be as low as 0.1 mBq/kg. A sample size of 10 g will be required to analyze such a dilute sample.

**Figure 5:**
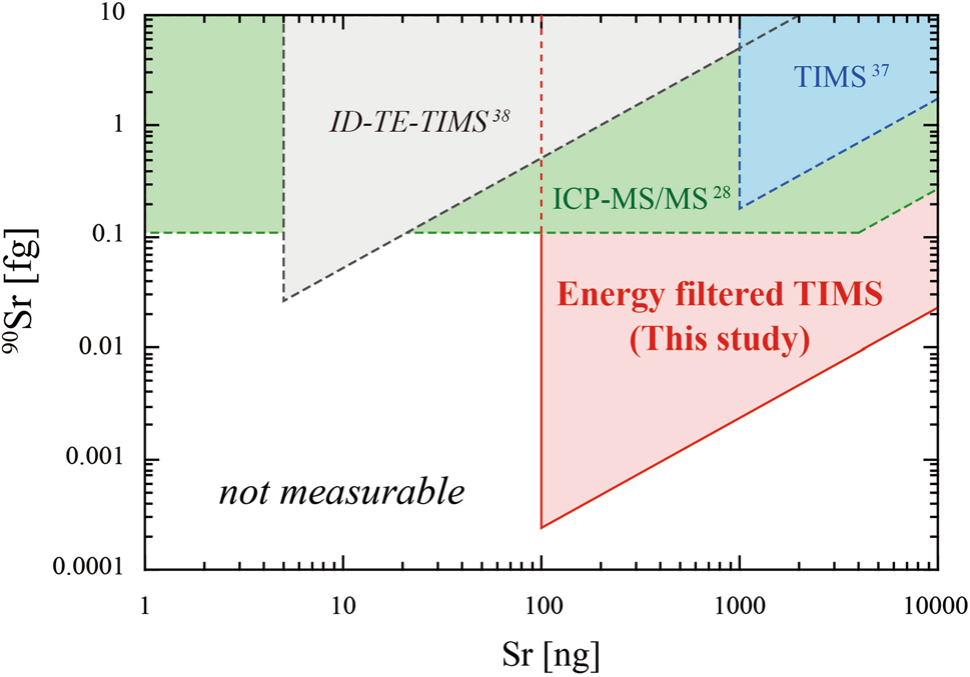
The capability of the mass spectrometric ^90^Sr determination methods is demonstrated using the absolute amount of stable Sr and ^90^Sr required for a single measurement. Samples within the shaded area are measured by the method. The lowest point of the shaded area corresponds to the detection limit of ^90^Sr for TIMS.

Comparing the capability of ^90^Sr analysis between TIMS and ICP-MS is not straightforward. The capability of ICP-MS is limited by the detection limit of the absolute amount of ^90^Sr and the abundance sensitivity defined as the ^90^Sr/^88^Sr ratio (Fig. 5). It is not limited by the amount of stable Sr unless the ^90^Sr/^88^Sr ratio was interfered with the abundance sensitivity limit. However, the capability of TIMS is limited by the detection limit of ^90^Sr/^88^Sr ratio and the minimum amount of sample required for the analysis (Fig. 5). The latter is required to keep the fixed ion beam intensity of ^88^Sr during the measurement because the performance of the ^90^Sr/^88^Sr ratio measurement by TIMS is mainly determined by the ion beam intensity of the most abundant ^88^Sr. The detection limit of the absolute amount of ^90^Sr is a derivative of these two parameters for TIMS. Compared with the previous TIMS and ICP-MS/MS techniques, a significant reduction in the ^90^Sr/^88^Sr detection limit achieved in this study had widely expanded the applicability of this technique to analyze samples with low ^90^Sr contents (Fig. 5). Additionally, an order of magnitude reduction of the minimum sample amount compared with the previous TIMS technique allows the analysis of 10 times smaller sample sizes. These features are advantageous in analyzing trace amounts of ^90^Sr on size-limited environmental samples such as soil exchangeable fraction and eolian dust as well as biological samples with low Sr abundances.

## Methods

### Reagents and standards

For sample preparation, ultrahigh purity grade acids, HNO_3_, HCl, HClO_4_, and HF (TAMAURE AA-100; Tama Chemicals Co., Ltd, Japan.), as well as ultra-pure water produced by a Milli-Q Element system (Millipore, USA) were used. The Sr isotopic reference material SRM 987 issued by NIST was used as a ^90^Sr-free reference standard.

### Sample preparation

The IAEA reference material’s certified ^90^Sr activity values were calculated using their dry weight. Therefore, these materials were weighed and digested as they were. Concentrated HNO_3_ and HClO_4_ acids were used to digest IAEA 156 and IAEA 330. The Fukushima samples were analyzed on an ashed sample, which were used in the previous ^90^Sr activity analysis^20^. The ashed crayfish and smallmouth bass samples were weighed and digested successively using 2.4 M HCl and conc HNO_3_. JCp-1 was digested with 5% CH_3_COOH. Finally, all the samples were dissolved in 3 M HNO_3_. NASS-6 was mixed with 6 M HNO_3_ to make a 3 M HNO_3_ solution.

Sr was separated from the other elements, including Zr, using extraction chromatography with Sr Resin (Eichrom Technologies Inc., USA) using a handmade PTFE column of 0.2 mL volume^34^. After sample solution loading, the column was rinsed with 2 mL of 6 M HNO_3_ and 0.5 mL of 3 M HNO_3_ successively, and Sr was eluted with 2 mL of 0.05 M HNO_3_. Finally, the separated Sr fraction was reacted with one drop of concentrated HNO_3_ to decompose resin-derived organics. The total yield of the Sr separation chemistry was approximately 94%^34^. After the Sr separation chemistry, the Zr/Sr ratio was reduced to 1.2 × 10^–5^ times the original ratio of the sample.

### Quantitative analysis of Sr and the reference ^90^Sr/^88^Sr ratio of the samples

The Sr concentrations of the ^90^Sr containing samples (IAEA 156, IAEA 330, crayfish, and smallmouth bass) were measured using ICP-MS Agilent 7700 (Agilent Technologies, Japan) and a sample of a fraction of the sample solution at Kochi Institute for Core Sample Research, JAMSTEC. The measured Sr concentration was used to calculate the reference ^90^Sr/^88^Sr values.

### Instrumentation

In this study, two thermal ionization mass spectrometers were used, the Triton™ XT (Thermo Scientific™) at Thermo Fisher Scientific, Bremen, Germany, and the Triton™ *Plus* (Thermo Scientific™) at Fukushima University, Japan. Both instruments are equipped with eight movable Faraday cups, an axial Faraday cup, and an axial secondary electron multiplier (SEM). The Faraday cups are connected with the standard 10^11^ Ω amplifiers. A set of RPQ lenses, which is an energy filtering device, is equipped in front of the SEM detector. The configuration of the detectors was summarized in Table S4 in supporting information. The peak position of ^88^Sr and ^90^Sr with this collector setting was shown in Figure S7 in supporting information.

This study uses both Re and Re-ZR (rhenium zone-refined) single filaments. High temperature bakeout of the filaments at 5.5 A filament current were performed under high vacuum for 30 min (Bremen) or 60 min (Fukushima). In Fukushima, the baked filaments are further inspected for ^90^Zr emission. ^90^Zr intensity of the baked filaments is measured at filament temperature of approximately 1550 °C for 1200 s. Filaments with ^90^Zr intensities higher than 0.04 cps are disposed of at this point.

### ^90^Sr/^88^Sr ratio measurement by TIMS

The separated sample containing 100–400 ng of Sr was loaded onto a baked filament with a Ta activator. The amount of Sr in the samples was fixed just before the Sr separation. The actual sample amount on the filament is lower than this nominal amount because losses of sample during the Sr separation and sample loading are not included in this number. In Fukushima, dull-red heating during sample loading was avoided because the sample becomes fragile and tends to be partially lost from the filament surface. Maximum Sr ion yield of the analytical condition in Fukushima, estimated by loading 108 ng NIST SRM987 on the filament and keeping the ^88^Sr signal of 25 V until sample exhaustment, was 2.1% (Table S5 in the supporting information). In both laboratories, Sr isotopes are measured using static multicollection mode with the slightly shifted axial mass settings of 89.8777 to eliminate the isobaric interference of organic molecules on m/z 90.0^38^. In Bremen, filaments were initially heated to 2400 mA at a rate of 300 mA/min while measuring a 5-min baseline for Faraday cup detectors. The filament current increased at 100 mA/min until an ^88^Sr signal of 20 V was achieved. After a 1-min wait, the filament current was re-adjusted and the measurement was initiated. Ion beam integration of 4.194 s was repeated for approximately 1 h (10 blocks, 86 cycles per block). During the measurement, the filament current was adjusted to keep the ^88^Sr signal within 20 ± 4 V. In Fukushima, filaments were preheated at 2250 mA for 30 min before the measurement. During the measurement, filaments were heated to 1500 mA at a rate of 1500 A/min and then to 2250 mA at a rate of 214 mA/min. The filament current was increased at 100 mA/min until an ^88^Sr signal of 25 V was achieved. Ion beam integration of 16.777 s was repeated for approximately 1 h (22 blocks, 10 cycles per block). During the measurement, the inter-block filament current adjustment kept the ^88^Sr signal at ± 2.5 V. The Faraday cup baseline was measured using a long baseline method utilizing the very stable nature of the Faraday cup noise level of TRITON^41,42^. A 1 h baseline was measured for every 10 h of the measurement sequence. After the first analytical session in Fukushima, the operating voltage of the SEM detector was adjusted, resulting in slightly different analytical conditions between the two sessions. In both laboratories, FC-SEM yield was calibrated just before the measurement using ^84^Sr ion beam of ca. 5 × 10^−14^ A (ca. 3 × 10^5^ cps). Both the RPQ transmission efficiency and the SEM counting efficiency was corrected by this FC-SEM yield. Typical vacuum condition of the analyzer chamber during the measurement were 1.5 × 10^−9^ mbar and 4.5 × 10^−9^ mbar for Bremen and Fukushima instruments, respectively. The dark noise of the SEM detector was measured for 1 h along with the Faraday cup baselines. However, all the measurements are not corrected for dark noise. Instead, the average value of multiple ^90^Sr-free NIST 987 measurements was used to correct overall noise signals on ^90^Sr. The analytical error of the ^90^Sr/^88^Sr ratio was determined as the 2σ internal error, which is two times the standard error of the ^90^Sr/^88^Sr ratios of 220 or 860 cycles. In all the measurements, the analytical error of the ^90^Sr/^88^Sr ratio was comparable with the √n counting error of the accumulated ^90^Sr count before noise correction.

Instrumental isotope fractionation is a possible error source of the ^90^Sr/^88^Sr ratio measured using mass spectrometry. However, the variability of the average ^88^Sr/^86^Sr values caused by the instrumental mass fractionation during the measurement was ± 0.5% (^88^Sr/^86^Sr values from 8.31 to 8.41) and was negligible compared with the analytical error of ^90^Sr/^88^Sr ratio (> 10%, 2RSD) in this study. Therefore, no correction for instrumental isotope fractionation was made.

Concentration of Zr in NIST 987 and Ta-activator solutions were measured ICP-MS with a dilution ratio of 10,000. For both reagents, Zr concentrations were under detection limit, which limits the Zr concentration of the original solutions as < 1.6 × 10^2^ ppb.

### Noise correction scheme for ^90^Sr signal

The stable nature of the noise signal allows precise noise correction on ^90^Sr measurement. Our noise correction scheme is as follows. First, the average ^90^Sr noise signal value was determined using multiple measurements of ^90^Sr-free NIST SRM-987. Then, the value is subtracted from ^90^Sr intensities of every measurement cycle in the run. All the data in the measurement were re-calculated off-line using the noise corrected ^90^Sr intensities, and finally, the noise corrected ^90^Sr/^88^Sr ratios are determined. The correction value should be determined every analytical session because the noise intensity level is sensitive to the SEM parameters and analytical conditions. A single correction value is used for all measurements within the analytical session.

## Conclusions

This study demonstrates the ability of a TIMS instrument to perform an accurate ^90^Sr/^88^Sr ratio measurement of samples with ^90^Sr/^88^Sr ratios as low as 2.7 × 10^−12^. Not only the introduction of effective hardware such as the RPQ lenses but a thorough investigation and elimination of noise signals have resulted in a constantly low noise signal of ^90^Sr on the 0.001 cps order. The sample throughput of this technique was 40 samples per day for Sr separation chemistry and 21 samples per 2 days for isotope ratio measurement. This is not as fast as the ICP-MS techniques but is significantly faster than radiometric methods. To detect 0.0012 mBq of ^90^Sr, this energy-filtered TIMS technique requires a sample size of 100 ng of Sr. This sample size corresponds to 12.3 µL of seawater or less than 10 mg of biological samples with Sr abundances larger than 10 mg/kg, which seems easy to handle. The lowered ^90^Sr detection limit and smaller sample size of this technique are suitable for studying environmental diffusion of radioactive materials, as well as environmental elemental cycling studies using nuclear test origin ^90^Sr in the earth surface materials.

## Supplementary Information

Supplementary Information.

## References

[1] Yoshida H, Asahara Y, Yamamoto K, Katsuta N, Minami M, Metcalfe R (2019). ^87^Sr/^86^Sr age determination by rapidly formed spherical carbonate concretions. Sci. Rep..

[2] Francisci G, Micarelli I, Iacumin P, Castorina F, Di Vincenzo F, Di Matteo M, Giostra C, Manzi G, Tafuri MA (2020). Strontium and oxygen isotopes as indicators of Longobards mobility in Italy: An investigation at Povegliano Veronese. Sci. Rep..

[3] Blum JD, Erel Y (1995). A silicate weathering mechanism linking increases in marine ^87^Sr/ ^86^Sr with global glaciation. Nature.

[4] Tipple BJ, Valenzuela LO, Ehleringer JR (2018). Strontium isotope ratios of human hair record intra-city variations in tap water source. Sci. Rep..

[5] Sun, X, Zhang, F, Gutiérrez-Gamboa, G, Ge, Q, Xu, P, Zhang, Q, Fang, Y., & Ma, T. Real wine or not? Protecting wine with traceability and authenticity for consumers: chemical and technical basis, technique applications, challenge, and perspectives. *Crit. Rev. Food Sci. Nutr.* 1–27 (2021). 10.1080/10408398.2021.1906624 10.1080/10408398.2021.1906624 33825545

[6] DeBord, J. S. Predicting the geographic origin of heroin by multivariate analysis of elemental composition and strontium isotope ratios. (2018). 10.25148/etd.FIDC006831

[7] Sahoo SK, Kavasi N, Sorimachi A, Arae H, Tokonami S, Mietelski JW, Łokas E, Yoshida S (2016). Strontium-90 activity concentration in soil samples from the exclusion zone of the Fukushima daiichi nuclear power plant. Sci. Rep..

[8] The Japan Chemical Analysis Center. The Environmental Radiactivity and Radiation in Japan (Database). *Nucl. Regul. Auth.* http://www.kankyo-hoshano.go.jp/en/

[9] Konno M, Takagai Y (2018). Determination and comparison of the Strontium-90 concentrations in topsoil of Fukushima prefecture before and after the Fukushima Daiichi nuclear accident. ACS Omega.

[10] Feuerstein J, Boulyga SFF, Galler P, Stingeder G, Prohaska T (2008). Determination of ^90^Sr in soil samples using inductively coupled plasma mass spectrometry equipped with dynamic reaction cell (ICP-DRC-MS). J. Environ. Radioact..

[11] Saniewski M, Zalewska T, Krasińska G, Szylke N, Wang Y, Falandysz J (2016). ^90^Sr in King Bolete Boletus edulis and certain other mushrooms consumed in Europe and China. Sci. Total Environ..

[12] Steinhauser G, Brandl A, Johnson TE (2014). Comparison of the Chernobyl and Fukushima nuclear accidents: a review of the environmental impacts. Sci. Total Environ..

[13] Steinhauser G, Schauer V, Shozugawa K (2013). Concentration of Strontium-90 at Selected Hot Spots in Japan. PLOS ONE.

[14] Konno M, Takagai Y (2018). Simple radiometric determination of Strontium-90 in seawater using measurement of Yttrium-90 decay time following iron-barium Co-precipitation. Anal. Sci..

[15] Furukawa M, Takagi K, Matsunami H, Komatsuzaki Y, Kawakami T, Shinano T, Takagai Y (2019). Rapid quantification of radioactive Strontium-90 in fresh foods via online solid-phase extraction-inductively coupled plasma-dynamic reaction cell-mass spectrometry and its comparative evaluation with conventional radiometry. ACS Omega.

[16] Ministry of Health Labour and Welfare (Japan). Inspection of Radiomaterials in Tap water. (2011). http://www.mhlw.go.jp/file/06-Seisakujouhou-10900000-Kenkoukyoku/01_houshasei_120328_m1.pdf

[17] Fukushima prefecture Government. Results of Environmental Radioactivity Monitoring. (2011). http://www.pref.fukushima.lg.jp/sec_file/monitoring/k-1/kaisui110516-110530.pdf

[18] Koarai K, Kino Y, Takahashi A, Suzuki T, Shimizu Y, Chiba M, Osaka K, Sasaki K, Fukuda T, Isogai E, Yamashiro H, Oka T, Sekine T, Fukumoto M, Shinoda H (2016). ^90^Sr in teeth of cattle abandoned in evacuation zone: Record of pollution from the Fukushima-Daiichi nuclear power plant accident. Sci. Rep..

[19] Furukawa, M. & Takagai, Y. *Agricultural Implications of the Fukushima Nuclear Accident (III)*. *Agric. Implic. Fukushima Nucl. Accid.* (Springer Singapore, 2019). 10.1007/978-981-13-3218-0

[20] MEXT Research and Development Bureau Atomic Energy Division. *Analytical methods of the radioactive strontium, No.2*. *Minist. Educ. Cult. Sport. Sci. Technol. Japan* (2003). http://www.kankyo-hoshano.go.jp/series/lib/No2.pdf

[21] Kameo Y, Katayama A, Fujiwara A, Haraga T, Nakashima M (2007). Rapid determination of ^89^Sr and ^90^Sr in radioactive waste using Sr extraction disk and beta-ray spectrometer. J. Radioanal. Nucl. Chem..

[22] Amano H, Sakamoto H, Shiga N, Suzuki K (2016). Method for rapid screening analysis of Sr-90 in edible plant samples collected near Fukushima Japan. Appl. Radiat. Isot..

[23] Tazoe H, Obata H, Yamagata T, Karube Z, Nagai H, Yamada M (2016). Determination of strontium-90 from direct separation of yttrium-90 by solid phase extraction using DGA Resin for seawater monitoring. Talanta.

[24] Firestone RB, Shirley VS (1996). Table of Isotopes (CD-ROM ver).

[25] L’Annunziata M (2012). Handbook of Radioactivity Analysis.

[26] Lehto J, Hou XJ (2011). Chemistry and Analysis of Radionuclides.

[27] Takagai Y, Furukawa M, Kameo Y, Suzuki K (2014). Sequential inductively coupled plasma quadrupole mass-spectrometric quantification of radioactive strontium-90 incorporating cascade separation steps for radioactive contamination rapid survey. Anal. Methods.

[28] Ohno T, Hirono M, Kakuta S, Sakata S (2018). Determination of strontium 90 in environmental samples by triple quadrupole ICP-MS and its application to Fukushima soil samples. J. Anal. At. Spectrom..

[29] Furukawa M, Takagai Y (2016). Split flow online solid-phase extraction coupled with inductively coupled plasma mass spectrometry system for one-shot data acquisition of quantification and recovery efficiency. Anal. Chem..

[30] Furukawa M, Matsueda M, Takagai Y (2018). Ultrasonic mist generation assist argon-nitrogen mix gas effect on radioactive strontium quantification by online solid-phase extraction with inductively coupled plasma mass spectrometry. Anal. Sci..

[31] Yanagisawa K, Matsueda M, Furukawa M, Takagai Y (2020). Development of online dilution system for quantification of ^90^Sr using automatic solid-phase extraction inductively coupled plasma mass spectrometry. Anal. Sci..

[32] Eiden GC, Barinaga CJ, Koppenaal DW (1997). Beneficial ion/molecule reactions in elemental mass spectrometry. Rapid Commun. Mass Spectrom.

[33] Favre G, Brennetot RR, Chartier F, Vitorge P (2007). Understanding reactions with O_2_ for ^90^Sr measurements by ICP-MS with collision-reaction cell. Int. J. Mass Spectrom..

[34] Wakaki S, Obata H, Tazoe H, Ishikawa T (2017). Precise and accurate analysis of deep and surface seawater Sr stable isotopic composition by double-spike thermal ionization mass spectrometry. Geochem. J..

[35] Fukutani S, Fujii T, Yamana H (2008). Measurement of strontium isotopic ratio in natural matrix sample containing strontium-90. J. Nucl. Sci. Technol..

[36] Shibahara, Y., Kubota, T., Fukutani, S., Fujii, T., Takamiya, K., Ohta, T., Shibata, T., Yoshikawa, M., Konno, M., Mizuno, S. & Yamana, H. in *Radiological Issues for Fukushima’s Revitalized Future* 33–46 (Springer Japan, 2016). 10.1007/978-4-431-55848-4_4

[37] Kavasi N, Sahoo SK (2019). Method for ^90^Sr analysis in environmental samples using thermal ionization mass spectrometry with Daly ion-counting system. Anal. Chem..

[38] Ito C, Shimode R, Miyazaki T, Wakaki S, Suzuki K, Takagai Y (2020). Isotope dilution-total evaporation–thermal ionization mass spectrometric direct determination of radioactive Strontium-90 in microdrop samples. Anal. Chem..

[39] Kavasi N, Sahoo SK, Arae H, Aono T, Palacz Z (2019). Accurate and precise determination of ^90^Sr at femtogram level in IAEA proficiency test using thermal ionization mass spectrometry. Sci. Rep..

[40] van Calsteren P, Schwieters JB (1995). Performance of a thermal ionisation mass spectrometer with a deceleration lens system and post-deceleration detector selection. Int. J. Mass Spectrom. Ion Process..

[41] Koornneef JM, Bouman C, Schwieters JB, Davies GR (2013). Use of 10^12^ ohm current amplifiers in Sr and Nd isotope analyses by TIMS for application to sub-nanogram samples. J. Anal. At. Spectrom..

[42] Wakaki S, Ishikawa T (2018). Isotope analysis of nanogram to sub-nanogram sized Nd samples by total evaporation normalization thermal ionization mass spectrometry. Int. J. Mass Spectrom..

