# Author Correction: A part per trillion isotope ratio analysis of ^90^Sr/^88^Sr using energy-filtered thermal ionization mass spectrometry

**DOI:** 10.1038/s41598-022-14464-8

**Published:** 2022-06-09

**Authors:** Shigeyuki Wakaki, Jo Aoki, Ryoya Shimode, Katsuhiko Suzuki, Takashi Miyazaki, Jenny Roberts, Hauke Vollstaedt, Satoshi Sasaki, Yoshitaka Takagai

**Affiliations:** 1 Kochi Institute for Core Sample Research, Japan Agency for Marine-Earth Science and Technology (JAMSTEC), 200 Monobe Otsu, Nankoku, Kochi 783-8502 Japan; 2 Faculty of Symbiotic Systems Science, Cluster of Science and Technology, Fukushima University, 1 Kanayagawa, Fukushima, 960-1296 Japan; 3 Submarine Resources Research Center, JAMSTEC, 2-15 Natushima, Yokosuka, Kanagawa 237-0061 Japan; 4 Volcanoes and Earth’s Interior Research Center, JAMSTEC, 2-15 Natushima, Yokosuka, Kanagawa 237-0061 Japan; 5 Thermo Fisher Scientific Bremen GmbH, Hanna-Kunath-Str. 11, 28199 Bremen, Germany; 6 Thermo Fisher Scientific K.K, 4-2-8 Shibaura, Minato-ku, Tokyo 208-0023 Japan; 7 Institute of Environmental Radioactivity, Fukushima University, 1 Kanayagawa, Fukushima, 960-1296 Japan

Correction to: *Scientific Reports* 10.1038/s41598-022-05048-7, published online 21 January 2022

The original version of this Article contained an error in Table 1 where row 2 was incorrect for “^90^Sr/^88^Sr^1,2^ Abundance sensitivity”,

“1.0 (1.8) × 10^−11^”.

now reads:

“1.0 (2.2) × 10^−11^”.

The original Article has been corrected.

