# Supplementary material for: A part per trillion isotope ratio analysis of 90Sr/88Sr using energy-filtered thermal ionization mass spectrometry
Source: Sci Rep. 2022 Jan 21;12:1151. doi: 10.1038/s41598-022-05048-7 (PMC8783016; doi:10.1038/s41598-022-05048-7)
Supplement: Supplementary file 1 — Supplementary Information. [file 41598_2022_5048_MOESM1_ESM.docx]

Supporting Information for *Scientific Reports*

A Part Per Trillion Isotope Ratio Analysis of ^90^Sr/^88^Sr using Energy-Filtered Thermal ionization mass spectrometry

Shigeyuki Wakaki^1†^, Jo Aoki^2†^, Ryoya Shimode^2^, Katsuhiko Suzuki^3†^, Takashi Miyazaki^4†^, Jenny Roberts^5^, Hauke Vollstaedt^5^, Satoshi Sasaki^6^ and Yoshitaka Takagai^2,7†*^

1 Kochi Institute for Core Sample Research, Japan Agency for Marine-Earth Science and Technology (JAMSTEC), 200 Monobe Otsu, Nankoku, Kochi 783-8502, JAPAN

2 Faculty of Symbiotic Systems Science, Cluster of Science and Technology, Fukushima University, 1 Kanayagawa, Fukushima 960-1296, Japan

3 Submarine Resources Research Center, JAMSTEC, 2-15 Natushima, Yokosuka, Kanagawa 237-0061, Japan

4 Volcanoes and Earth’s Interior Research Center, JAMSTEC, 2-15 Natushima, Yokosuka, Kana-gawa 237-0061, Japan

5 Thermo Fisher Scientific Bremen GmbH, Hanna-Kunath-Str. 11, Bremen 28199, Germany

6 Thermo Fisher Scientific K.K, 4-2-8 Shibaura, Minato-ku, Tokyo 208-0023, Japan

7 Institute of Environmental Radioactivity, Fukushima University, 1 Kanayagawa, Fukushima, 960-1296 Japan.

*To whom correspondence should be addressed: E-mail: s015@ipc.fukushima-u.ac.jp

^†^ These authors contributed equally to this work

**Table of Contents**

Section Page

1. The effect of RPQ on signal and noise intensities (Figure S1) S2
2. ^88^Sr peak tail intensities on m/z = 90 (Figure S2) S3
3. Mass spectrum in the vicinity of m/z = 90 (Figure S3) S4
4. Transition of Noise signal during Sr isotope ratio measurement (Figure S4) S5
5. Measured ^90^Sr/^88^Sr ratios of NIST SRM987 (Figure S5) S6
6. ^90^Sr activity parameters of the environmental samples (Table S1) S7
7. Summary of ^90^Sr/^88^Sr ratios of the environmental samples (Table S2) S8
8. Results of ^90^Sr/^88^Sr ratios of the environmental samples (Table S3) S9
9. Measured ^90^Sr/^88^Sr ratios of the environmental samples (Figure S6) S11
10. Detector configuration of TIMS measurements (Table S4) S12
11. Peak position of ^90^Sr and ^88^Sr during analysis (Figure S7) S13
12. Estimated Sr ion yield (Table S5) S14

**
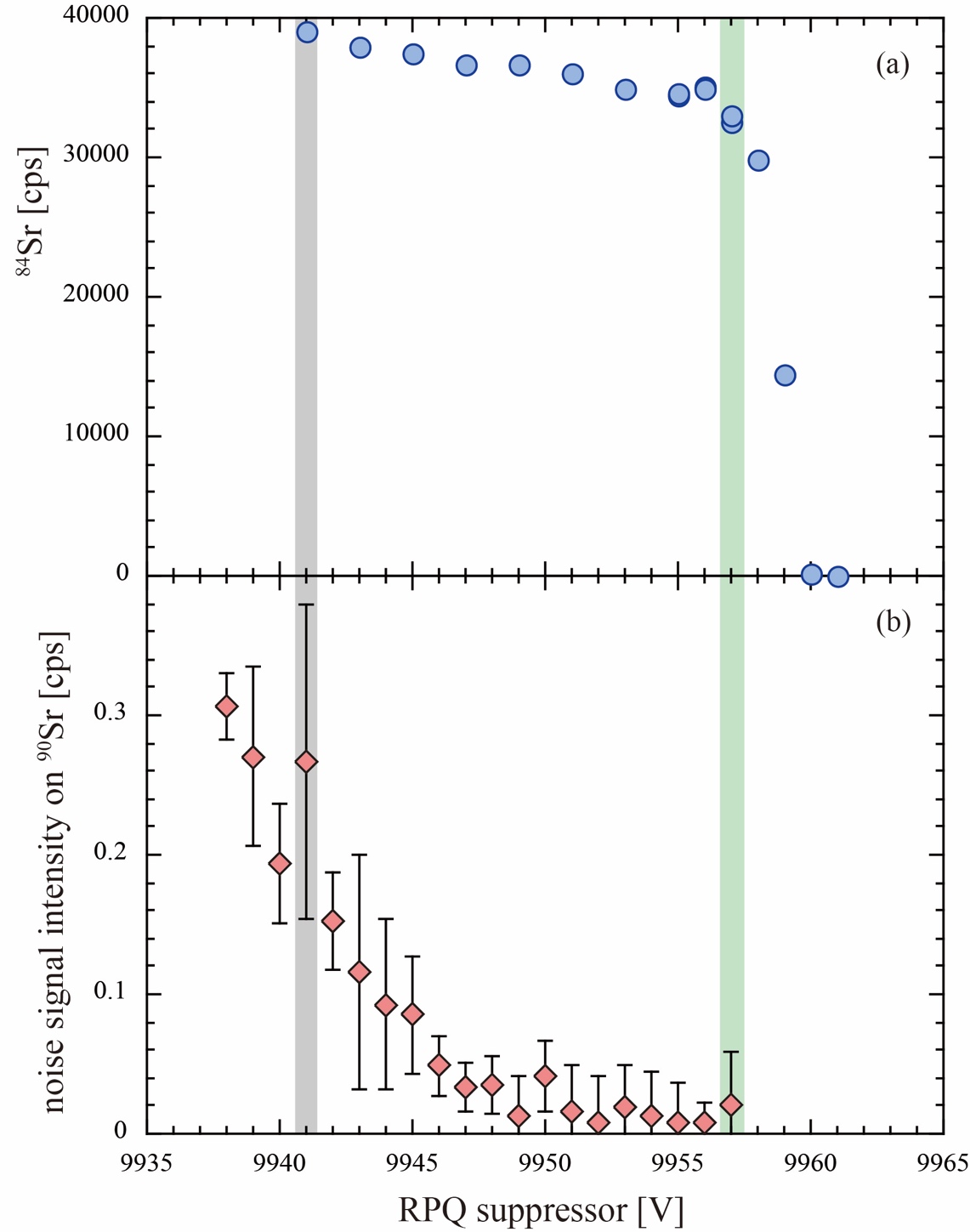
**

**Figure S1.** The effect of RPQ parameter on (a) ^84^Sr signal intensity, and (b) ^88^Sr peak tail intensity on ^90^Sr. In both measurements, NIST 987 was used as a sample. The ^84^Sr signal was measured while keeping the ^88^Sr signal of 0.1V. The ^88^Sr peak tail signal was measured while keeping the ^88^Sr signal of 25V. For the peak tail measurement, each plot represents average of the 3 repeated measurement of 100 cycles of 16.777s integration. As the effect of RPQ gets stronger, the noise reduction efficiency increases but the ion transmission efficiency decreases. The suppressor parameter was determined by these data to obtain high noise reduction with moderate transmission efficiency decrease. The black band and the green band indicate the initial and the fine-tuned suppressor values of 9941 V and 9957 V, respectively.


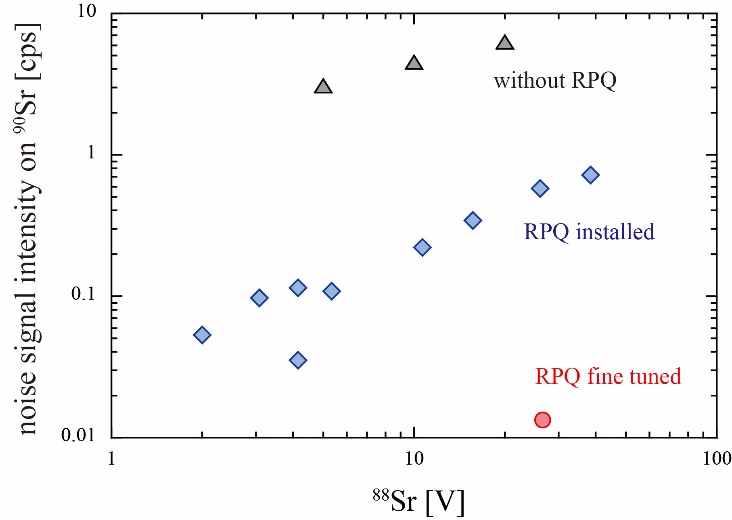


**Figure S2.** Noise signal intensity on ^90^Sr was plotted against the intensity of ^88^Sr. All data were taken using a sample of ^90^Sr-free NIST SRM987. Black triangle represents the data taken without RPQ. The blue diamond represents the data taken with RPQ but without fine-tuning. Red circle represents the data taken with fine-tuned RPQ. The positive correlation of the data shows the effect of the ^88^Sr peak tail on ^90^Sr.

**
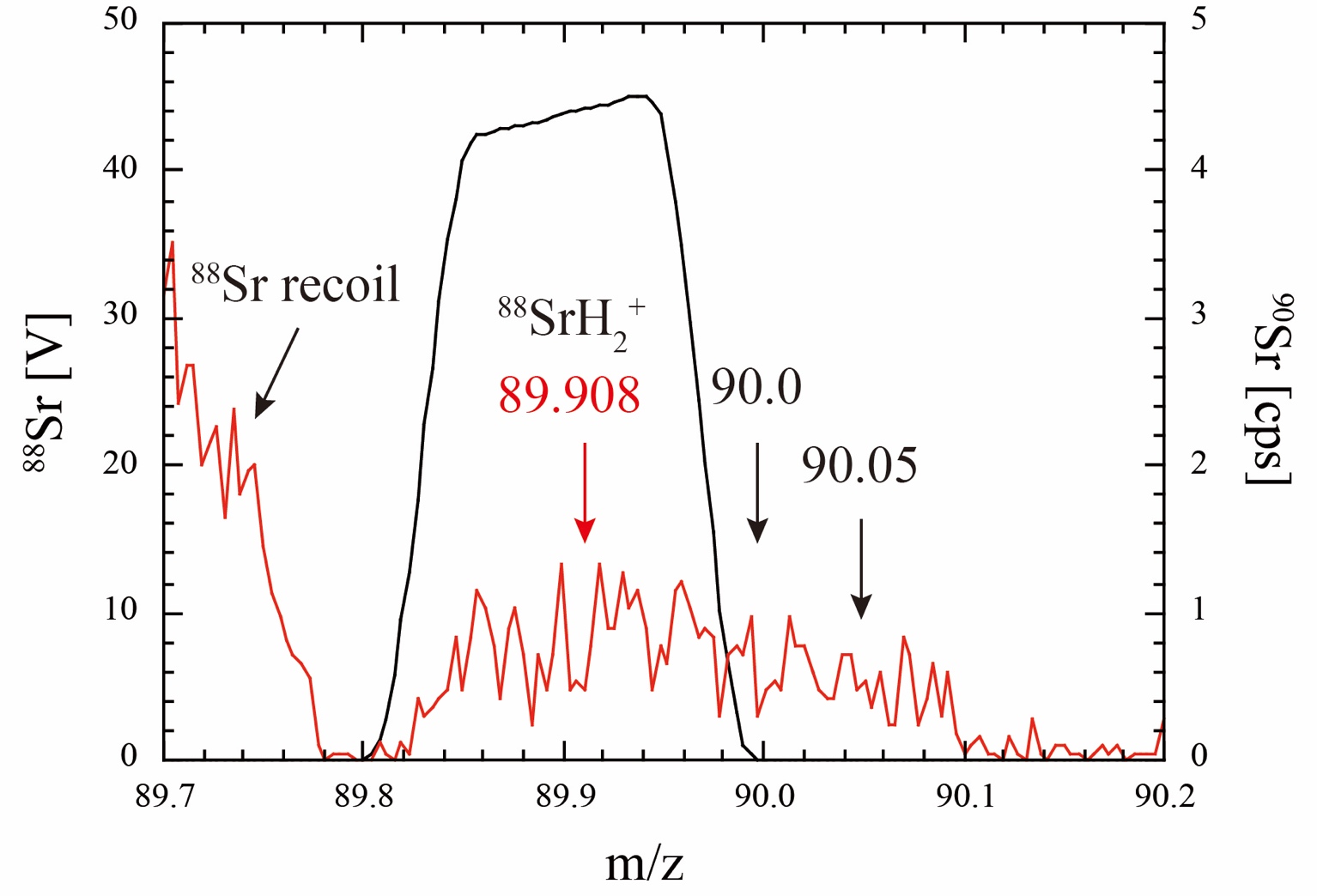
**

**Figure S3.** Mass spectrum of ^90^Sr noise observed in ^90^Sr-free NIST SRM987. To clearly show the noise spectrum, catheter (organics) was used upon sample loading and no pre-heating was made for this filament. The black line represents the ^88^Sr spectrum as measured by the L1 Faraday cup, and the red line represents the noise spectrum as measured by RPQ-SEM. The signal at m/z 89.7–89.78 corresponds to the recoil of the ^88^Sr ions in the collector housing. The formation of ^88^Sr recoil ions is owing to the geometry of the collector housing and the collector positions. Depending on the position of the large ^88^Sr beam in the collector array recoil ions might be produced when the beam is hitting apertures or Faraday cup housings. From m/z 89.70 to 89.78, the position of the ^88^Sr ion beam is between the center cup and the L1 cup. With such condition, ^88^Sr ions goes through the collector housing without hitting any faraday cup detectors and enters directly into the SEM flange, where the SEM detector is placed. By hitting the chamber walls of the SEM flange, ^88^Sr recoil ions are produced in the vicinity of the SEM detector. From m/z 89.78 to 90.2, the ^88^Sr ion beam is shielded by the L1 faraday cup detector. Because ^88^Sr ions do not enter the SEM flange, recoil ions are not produced under such condition. Note that ^88^Sr ions are totally collected in the L1 faraday cup detector at m/z = 89.8777. Therefore, such recoil ions are only apparent during mass scanning and are not produced during the ^90^Sr measurement. The intensity level of the ^88^Sr peak tail is represented by signals on m/z 89.78–89.82 and 90.1–90.2. With intensity levels lower than 1cps, the mass spectrum of ^88^SrH_2_^+^ with m/z 89.908 and those of the organics with m/z 90.0 and 90.05 were observed.


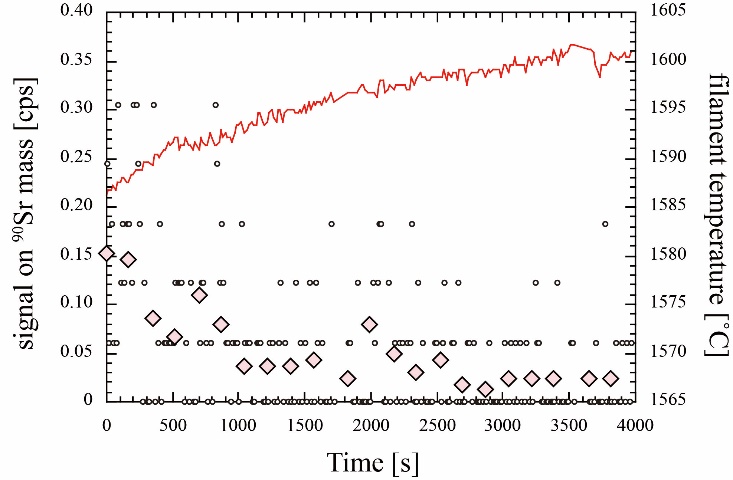


**Figure S4**. The transition of the noise signal intensities on ^90^Sr during measurement of ^90^Sr-free NIST SRM987 is plotted against the elapsed time. Small circles are raw data of each measurement cycle and pink diamonds are the average of 10 cycles. Changes in filament temperature during the measurement were plotted together in a red line.


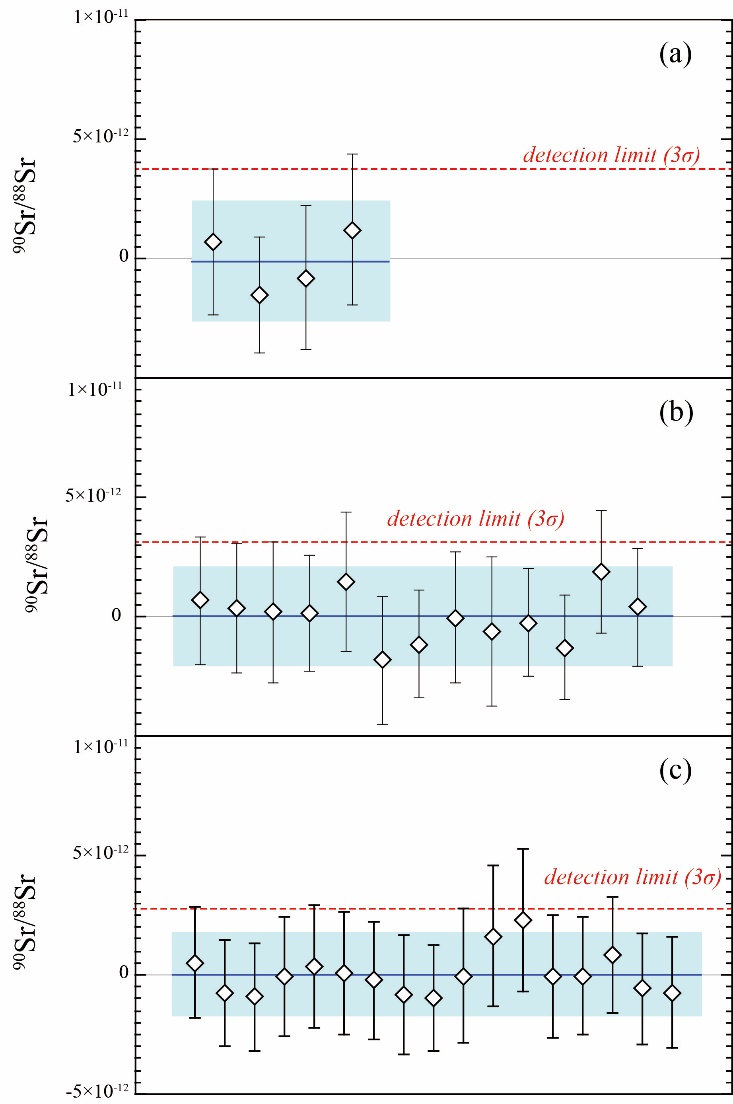


**Figure S5**. The noise corrected ^90^Sr/^88^Sr ratio of NIST SRM987 was measured in (a) Bremen, (b) Fukushima-1, and (c) Fukushima-2 sessions. White diamonds represent a single measurement. The blue line and blue band represent the average and 2 SD range of the multiple measurements. The red dotted line represents the detection limit of ^90^Sr/^88^Sr, defined as the 3-standard deviation of the multiple NIST SRM987 measurements.

**Table S1**. Summary of literature ^90^Sr activity parameters of the environmental samples^1)^.

| sample | sample type | ^90^Sr | Sr | status of | reference date |
| --- | --- | --- | --- | --- | --- |
|  |  | [Bq/kg] | [mg/kg] | ^90^Sr activity | for ^90^Sr activity |
| NASS-6 | seawater | ― | 7.48^2)^ | ― | ― |
| JCp-1 | coral | ― | 7271 (218) | ― | ― |
| IAEA 156 | clover | 14.8 (3.0) | 33.5 (2.0) | recommended value | August 1 1986 |
| IAEA 330 | spinach | 20.1 (4.2) | 44.0 (2.6) | certified value | October 15 2007 |
| Ash 1 | Crayfish | 110 (8) | 1344 (66) | measured value | July 12 2013 |
| Ash2 | Smallmouth bass | 25 (2) | 414 (28) | measured value | July 12 2013 |

1. All the errors in the parenthesis are 2SD.
2. Wakaki, S.; Obata, H.; Tazoe, H.; Ishikawa, T. Precise and Accurate Analysis of Deep and Surface Seawater Sr Stable Isotopic Composition by Double-Spike Thermal Ionization Mass Spectrometry. *Geochem. J.* **2017**, *51* (3), 227–239. https://doi.org/10.2343/geochemj.2.0461.

**Table S2**. Summarized results of ^90^Sr/^88^Sr measurements of the environmental samples.

| sample | session | reference date | reference ^90^Sr/^88^Sr ^1)^ | ^88^Sr [V] | ^90^Sr [cps] ^2)^ | ^90^Sr/^88^Sr ^2)^ | number of analysis |
| --- | --- | --- | --- | --- | --- | --- | --- |
| NASS-6 | *Fukushima-2* | *June 5th, 2021* | ― | 25.3 | 0.0000 (37) | -0.1 (2.5) × 10^-12^ | 5 |
| JCp-1 | *Fukushima-1* | *April 3rd, 2021* | ― | 26.9 | 0.0003 (48) | 0.3 (2.9) × 10^-12^ | 8 |
|  | *Fukushima-2* | *June 5th, 2021* | ― | 25.3 | 0.0008 (54) | 0.5 (3.4) × 10^-12^ | 5 |
| IAEA 156 | *Fukushima-2* | *June 5th, 2021* | 4.4 (0.9) × 10^-11^ | 26.8 | 0.0546 (51) | 3.26 (0.30) × 10^-12^ | 6 |
| IAEA 330 | *Bremen* | *January 22nd, 2020* | 7.9 (1.7) × 10^-11^ | 20.6 | 0.0886 (192) | 6.7 (1.3) × 10^-11^ | 4 |
|  | *Fukushima-2* | *June 5th, 2021* | 7.6 (1.7) × 10^-11^ | 25.9 | 0.0928 (43) | 5.76 (0.43) × 10^-11^ | 5 |
| Crayfish | *Fukushima-1* | *April 3rd, 2021* | 1.57 (0.15) × 10^-11^ | 28.0 | 0.0241 (90) | 1.38 (0.53) × 10^-11^ | 6 |
|  | *Fukushima-2* | *June 5th, 2021* | 1.56 (0.15) × 10^-11^ | 25.7 | 0.0210 (44) | 1.32 (0.42) × 10^-11^ | 6 |
| Smallmouth bass | *Fukushima-1* | *April 3rd, 2021* | 1.16 (0.11) × 10^-11^ | 27.9 | 0.0244 (71) | 1.40 (0.39) × 10^-11^ | 6 |
|  | *Fukushima-2* | *June 5th, 2021* | 1.15 (0.11) × 10^-11^ | 26.3 | 0.0217 (44) | 1.33 (0.27) × 10^-11^ | 6 |

All the errors in the parenthesis are 2SD.

1) Decay corrected to the reference date.

2) Noise corrected values using the average ^90^Sr count rate of NIST SRM 987 measurements.

**Table S3**. Detailed results of ^90^Sr/^88^Sr measurements of the environmental samples.

| sample | sample weight | session | Sr loaded | ^88^Sr^1)^ | ^90^Sr^1)^ | ^90^Sr/^88^Sr |
| --- | --- | --- | --- | --- | --- | --- |
|  | [mg] |  | [ng] | [V] | [cps] |  |
| NASS-6 | (0.06 mL) | *Fukushima-2* | 240 | 26.6 | 0.0005 (40) | 0.03 (0.24) × 10^-11^ |
|  | (0.06 mL) | *Fukushima-2* | 240 | 26.7 | -0.0016 (35) | -0.09 (0.21) × 10^-11^ |
|  | (0.06 mL) | *Fukushima-2* | 100 | 24.3 | 0.0029 (57) | 0.18 (0.38) × 10^-11^ |
|  | (0.06 mL) | *Fukushima-2* | 100 | 24.5 | -0.0001 (37) | -0.01 (0.26) × 10^-11^ |
|  | (0.06 mL) | *Fukushima-2* | 100 | 24.2 | -0.0016 (43) | -0.15 (0.28) × 10^-11^ |
| JCp-1 | 42.09 | *Fukushima-1* | 280 | 28.6 | -0.0024 (42) | -0.13 (0.24) × 10^-11^ |
|  |  | *Fukushima-1* | 280 | 27.8 | 0.0004 (46) | 0.02 (0.27) × 10^-11^ |
|  |  | *Fukushima-1* | 280 | 27.0 | 0.0045 (50) | 0.27 (0.30) × 10^-11^ |
|  |  | *Fukushima-1* | 280 | 28.0 | -0.0021 (45) | -0.12 (0.25) × 10^-11^ |
|  |  | *Fukushima-1* | 280 | 23.9 | 0.0012 (41) | 0.08 (0.27) × 10^-11^ |
|  |  | *Fukushima-1* | 280 | 27.2 | -0.0013 (40) | -0.07 (0.24) × 10^-11^ |
|  |  | *Fukushima-1* | 280 | 25.6 | 0.0026 (48) | 0.18 (0.32) × 10^-11^ |
|  |  | *Fukushima-1* | 280 | 27.2 | -0.0002 (44) | 0.00 (0.26) × 10^-11^ |
|  |  | *Fukushima-2* | 400 | 26.2 | -0.0004 (39) | -0.02 (0.24) × 10^-11^ |
|  |  | *Fukushima-2* | 100 | 25.4 | 0.0053 (65) | 0.33 (0.41) × 10^-11^ |
|  |  | *Fukushima-2* | 100 | 24.8 | -0.0004 (38) | -0.01 (0.25) × 10^-11^ |
|  |  | *Fukushima-2* | 100 | 25.1 | -0.0016 (42) | -0.11 (0.27) × 10^-11^ |
|  |  | *Fukushima-2* | 100 | 25.2 | 0.0010 (50) | 0.06 (0.32) × 10^-11^ |
| IAEA 156 | 30.76 | *Fukushima-2* | 275 | 27.2 | 0.0570 (100) | 3.36 (0.59) × 10^-11^ |
|  |  | *Fukushima-2* | 275 | 27.0 | 0.0549 (88) | 3.27 (0.53) × 10^-11^ |
|  | 34.05 | *Fukushima-2* | 275 | 26.0 | 0.0546 (99) | 3.37 (0.61) × 10^-11^ |
|  |  | *Fukushima-2* | 250 | 27.1 | 0.0575 (94) | 3.41 (0.56) × 10^-11^ |
|  | 31.40 | *Fukushima-2* | 275 | 26.9 | 0.0526 (84) | 3.11 (0.49) × 10^-11^ |
|  |  | *Fukushima-2* | 250 | 26.7 | 0.0508 (86) | 3.05 (0.52) × 10^-11^ |
| IAEA 330 | 32.21 | *Bremen* | 300 | 20.1 | 0.081 (10) | 6.44 (0.85) × 10^-11^ |
|  |  | *Bremen* | 300 | 20.1 | 0.092 (10) | 7.32 (0.89) × 10^-11^ |
|  | 34.93 | *Bremen* | 300 | 20.1 | 0.101 (11) | 7.04 (0.84) × 10^-11^ |
|  |  | *Bremen* | 300 | 21.9 | 0.081 (10) | 5.91 (0.78) × 10^-11^ |
|  | 41.69 | *Fukushima-2* | 310 | 26.7 | 0.094 (11) | 5.67 (0.65) × 10^-11^ |
|  |  | *Fukushima-2* | 310 | 27.0 | 0.091 (11) | 5.44 (0.68) × 10^-11^ |
|  | 51.56 | *Fukushima-2* | 100 | 24.8 | 0.090 (13) | 5.83 (0.87) × 10^-11^ |
|  |  | *Fukushima-2* | 100 | 25.0 | 0.094 (12) | 5.97 (0.80) × 10^-11^ |
|  |  | *Fukushima-2* | 100 | 25.9 | 0.095 (12) | 5.91 (0.76) × 10^-11^ |
| Crayfish | 44.67 | *Fukushima-1* | 400 | 28.3 | 0.0198 (70) | 1.12 (0.40) × 10^-11^ |
|  |  | *Fukushima-1* | 400 | 28.0 | 0.0256 (79) | 1.48 (0.46) × 10^-11^ |
|  | 37.93 | *Fukushima-1* | 400 | 27.8 | 0.0202 (66) | 1.17 (0.39) × 10^-11^ |
|  |  | *Fukushima-1* | 400 | 28.1 | 0.0320 (72) | 1.85 (0.42) × 10^-11^ |
|  |  | *Fukushima-1* | 400 | 28.0 | 0.0248 (75) | 1.41 (0.42) × 10^-11^ |
|  |  | *Fukushima-1* | 400 | 28.0 | 0.0225 (72) | 1.27 (0.41) × 10^-11^ |
|  |  | *Fukushima-2* | 200 | 26.5 | 0.0186 (66) | 1.13 (0.40) × 10^-11^ |
|  |  | *Fukushima-2* | 200 | 25.8 | 0.0174 (63) | 1.09 (0.40) × 10^-11^ |
|  |  | *Fukushima-2* | 100 | 25.5 | 0.0212 (60) | 1.34 (0.38) × 10^-11^ |
|  |  | *Fukushima-2* | 100 | 25.5 | 0.0253 (69) | 1.60 (0.44) × 10^-11^ |
|  |  | *Fukushima-2* | 100 | 25.6 | 0.0199 (70) | 1.24 (0.43) × 10^-11^ |
|  |  | *Fukushima-2* | 100 | 25.2 | 0.0236 (75) | 1.53 (0.48) × 10^-11^ |

**Table S3**. *Continued*

| sample | sample weight | session | Sr loaded | ^88^Sr^1)^ | ^90^Sr^1)^ | ^90^Sr/^88^Sr |
| --- | --- | --- | --- | --- | --- | --- |
|  | [mg] |  | [ng] | [V] | [cps] |  |
| Smallmouth bass | 58.61 | *Fukushima-1* | 400 | 27.7 | 0.0242 (77) | 1.40 (0.44) × 10^-11^ |
|  |  | *Fukushima-1* | 400 | 28.4 | 0.0303 (77) | 1.72 (0.44) × 10^-11^ |
|  |  | *Fukushima-1* | 400 | 27.9 | 0.0239 (76) | 1.37 (0.44) × 10^-11^ |
|  |  | *Fukushima-1* | 400 | 27.9 | 0.0228 (74) | 1.33 (0.42) × 10^-11^ |
|  | 26.57 | *Fukushima-1* | 400 | 28.0 | 0.0259 (69) | 1.48 (0.40) × 10^-11^ |
|  |  | *Fukushima-1* | 400 | 27.8 | 0.0195 (69) | 1.12 (0.39) × 10^-11^ |
|  |  | *Fukushima-2* | 200 | 26.4 | 0.0201 (67) | 1.22 (0.40) × 10^-11^ |
|  |  | *Fukushima-2* | 200 | 25.7 | 0.0215 (59) | 1.34 (0.37) × 10^-11^ |
|  |  | *Fukushima-2* | 200 | 26.5 | 0.0212 (67) | 1.29 (0.41) × 10^-11^ |
|  |  | *Fukushima-2* | 200 | 26.0 | 0.0224 (67) | 1.39 (0.42) × 10^-11^ |
|  |  | *Fukushima-2* | 200 | 26.4 | 0.0195 (63) | 1.18 (0.38) × 10^-11^ |
|  |  | *Fukushima-2* | 200 | 26.4 | 0.0256 (70) | 1.55 (0.43) × 10^-11^ |

All the errors in the parenthesis are 2SD.

1) Noise corrected values using the average ^90^Sr count rate of NIST SRM 987 measurements.

**
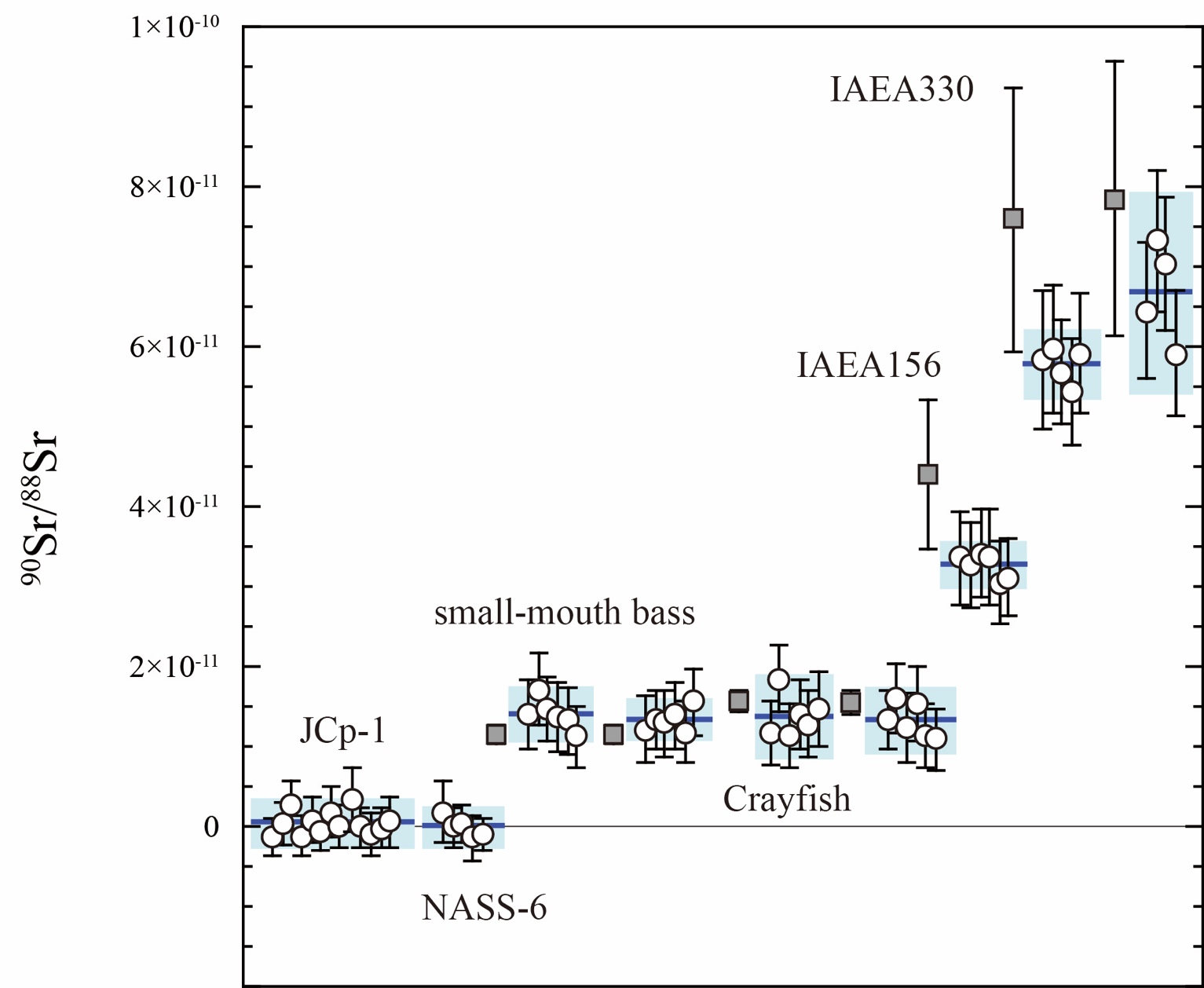
**

**Figure S6**. The noise corrected ^90^Sr/^88^Sr ratio of the environmental samples. White circles represent a single measurement. Blue lines and blue bands represent the average and 2 SD range of the multiple measurements. Grey square represents the decay corrected reference ^90^Sr/^88^Sr ratios.

Table S4 Detector configuration for ^90^Sr/^88^Sr measurement

| Collector | L4 | L3 | L2 | L1 | C |
| --- | --- | --- | --- | --- | --- |
| Isotope | ^84^Sr^*^ | ^86^Sr | ^87^Sr | ^88^Sr | ^90^Sr |
| Detector type | FC | FC | FC | FC | RPQ-SEM |

* ^84^Sr is not measured with TIMS TRITON XT at Bremen.


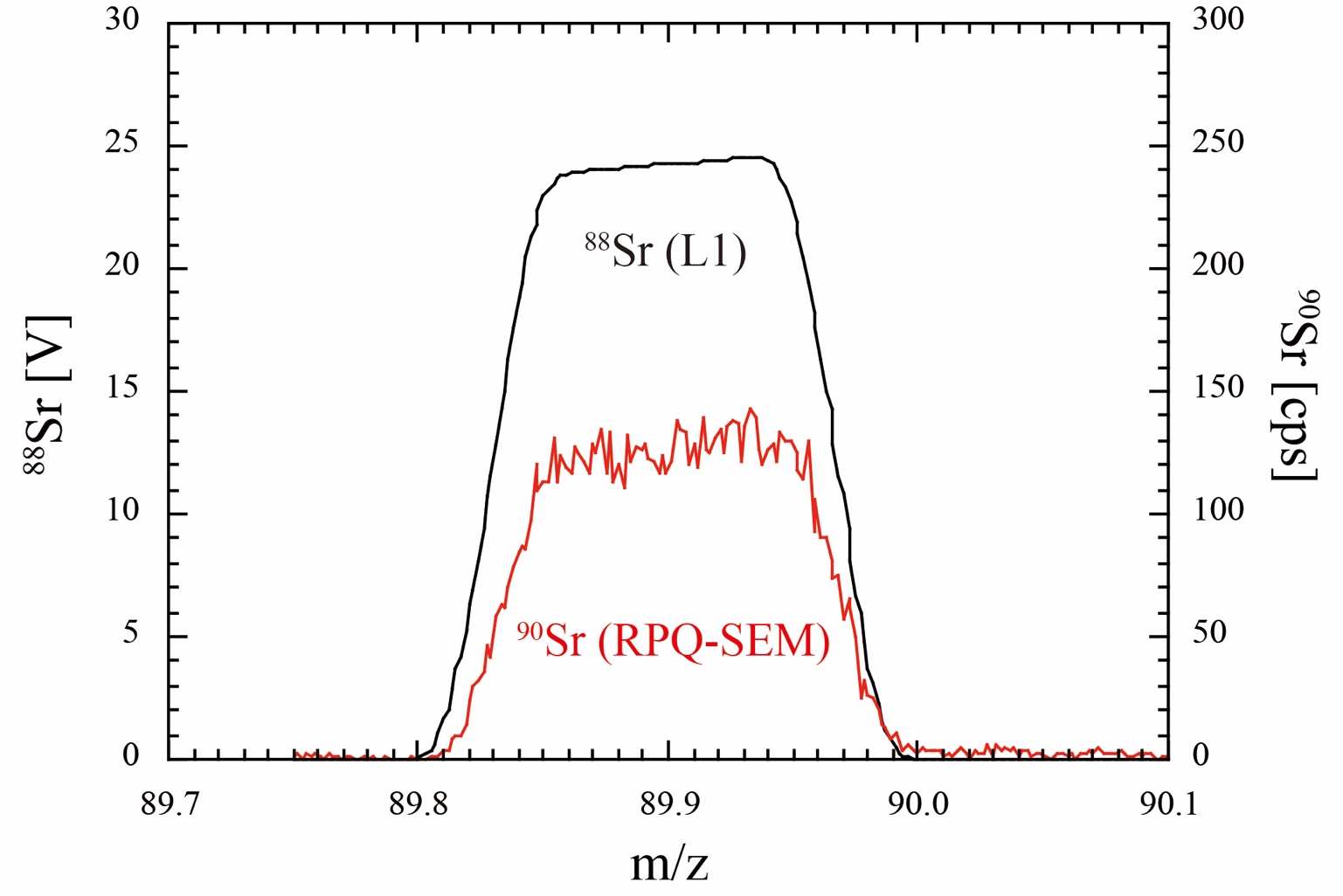


**Figure S7**. The mass spectrum of ^88^Sr (black line) and ^90^Sr (red line) showing the peak positions and collector settings. The spectrum was taken by ^90^Sr doped (ca. 100mBq) NIST SRM 987 sample. The perfect matche of the ^90^Sr spectrum to m/z 89.9077 demonstrates the accuracy of the mass calibration of our instrument.

**Table S5 Maximum Sr ion yield estimated from NIST SRM 987 analysis**

| sample | Sr loaded on the filament* | integrated ^88^Sr charge during warmup | integrated ^88^Sr charge during measurement | ^88^Sr collected in the detector | ion yield |
| --- | --- | --- | --- | --- | --- |
|  | [ng] | [As] | [As] | [ng] | [%] |
| SRM 987 | 108 | 1.5 × 10^-7^ | 2.13 × 10^-6^ | 2.51 | 2.32 |
| SRM 987 | 108 | 1.4 × 10^-7^ | 1.91 × 10^-6^ | 2.25 | 2.08 |
| SRM 987 | 108 | 1.4 × 10^-7^ | 1.69 × 10^-6^ | 2.01 | 1.86 |
| SRM 987 | 108 | 1.4 × 10^-7^ | 1.60 × 10^-6^ | 1.92 | 1.78 |
| SRM 987 | 108 | 1.4 × 10^-7^ | 2.08 × 10^-6^ | 2.44 | 2.26 |
| SRM 987 | 108 | 1.5 × 10^-7^ | 2.07 × 10^-6^ | 2.44 | 2.26 |
| SRM 987 | 108 | 1.4 × 10^-7^ | 1.79 × 10^-6^ | 2.12 | 1.96 |
| SRM 987 | 108 | 1.4 × 10^-7^ | 2.01 × 10^-6^ | 2.36 | 2.19 |
| SRM 987 | 108 | 1.4 × 10^-7^ | 1.56 × 10^-6^ | 1.87 | 1.73 |
| average |  |  |  | 2.23 | 2.05 |
| (2SD) |  |  |  | 0.52 | 0.45 |

All the samples were measured until the sample has exhaust.

Ion yield was calculated by the number of Sr ions counted in the detector against the number of Sr atoms loaded onto the filament.

***** A 1 μL of NIST SRM987 solution with Sr concentration of 108 ppm was loaded on the filament as a sample.
